# Distinct Profiles of Cell-Free MicroRNAs in Plasma of Veterans with Post-Traumatic Stress Disorder

**DOI:** 10.3390/jcm8070963

**Published:** 2019-07-03

**Authors:** Min Young Lee, David Baxter, Kelsey Scherler, Taek-Kyun Kim, Xiaogang Wu, Duna Abu-Amara, Janine Flory, Rachel Yehuda, Charles Marmar, Marti Jett, Inyoul Lee, Kai Wang, Leroy Hood

**Affiliations:** 1 Institute for Systems Biology, Seattle, WA 98109, USA; 2 Steven and Alexandra Cohen Veterans Center for Posttraumatic Stress and Traumatic Brain Injury, Department of Psychiatry, New York University, New York, NY 10003, USA; 3 Icahn School of Medicine at Mount Sinai, New York, NY 10029, USA; 4 James J. Peters VA Medical Center, The Bronx, NY 10468, USA; 5 Integrative Systems Biology, US Army Center for Environmental Health Research, Frederick, MD 21702, USA

**Keywords:** post-traumatic stress disorder, microRNA, next-generation sequencing, plasma, extracellular vesicles

## Abstract

Dysregulation of circulating microRNAs (miRNAs) in body fluids has been reported in psychiatric disorders such as schizophrenia, bipolar disorder, major depressive disorder, and post-traumatic stress disorder (PTSD). Recent studies of various diseases showed that extracellular vesicles (EV) in body fluids can provide different spectra of circulating miRNAs and disease-associated signatures from whole fluid or EV-depleted fraction. However, the association of miRNAs in EVs to PTSD has not been studied. In this study, we performed a comprehensive profiling of miRNAs in whole plasma, extracellular vesicles (EV) and EV-depleted plasma (EVD) samples collected from combat veterans with PTSD and matched controls by utilizing a next-generation sequencing (NGS) platform. In total, 520 circulating miRNAs were quantified from 24 male Iraq and Afghanistan combat veterans with (*n =* 12) and without (*n =* 12) PTSD. The overall miRNA profiles in whole plasma, EV and EVD fractions were different and miRNAs affected by PTSD were also distinct in each sample type. The concentration changes of miR-203a-3p in EV and miR-339-5p in EVD were confirmed in an independent validation cohort that consisted of 20 veterans (10 with and 10 without PTSD) using qPCR. The target genes of these two miRNAs were involved in signaling pathways and comorbid conditions associated with PTSD (e.g., neurotransmitter systems such as dopaminergic and serotonergic signaling, inflammatory response, and cardiovascular diseases). Our findings suggest that PTSD may have different impacts on miRNAs encapsulated in vesicles and outside of vesicles. Further studies using larger samples are needed to evaluate the utility of these miRNAs as diagnostic biomarkers for PTSD.

## 1. Introduction

Post-traumatic stress disorder (PTSD) poses a significant burden on emotional and physical health and health care costs [1,2]. According to the US Department of Veterans Affairs, about 12% of Gulf war veterans, including those deployed to Iraq and Afghanistan, suffer from PTSD in a given year and about 30% of Vietnam veterans have had PTSD in their lifetime [3,4,5]. The annual treatment cost for PTSD among service members is estimated to be approximately $1.6 billion [6]. The onset of PTSD is known to be influenced by various biological (e.g., glucocorticoid sensitivity and epigenetic modifications) and socio-economic risk factors (e.g., ethnicity and education levels) [1,7,8]. 

PTSD is a syndrome involving alteration of neuro-cognitive functions in brain. Emerging data suggests that this condition is a multisystem disorder affecting many biological systems including cardiovascular, liver metabolism, and immune system [1], raising the possibility that peripheral markers of illness may have utility in establishing the diagnosis of PTSD. At the current time, there are no validated biomarkers or laboratory tests that distinguish among trauma survivors with and without PTSD. Rather, diagnostic assessments are based on subjective clinical assessment, which can be affected by both over-reporting and under-reporting of symptoms. In addition, heterogeneity in clinical presentations of PTSD and overlapping symptoms with other conditions, such as traumatic brain injury and major depressive disorder, may mislead diagnosis and ultimately result in inappropriate treatment [9,10,11]. Therefore, objective biomarkers that can facilitate the process of diagnosing and differentiating PTSD are needed. 

MicroRNAs (miRNAs), a class of short noncoding regulatory RNAs, are one of the epigenetic modifiers of cellular gene expression and have been implicated in various biological processes [12]. Like other epigenetic modifiers including DNA methylation, miRNAs affect the gene expression level without altering genetic sequences [8]. In addition, miRNA have been observed in extracellular space and circulating in various body fluids, such as blood [13,14,15]. The association between miRNAs in body fluids and pathologic conditions has been reported in various diseases such as cancers [16,17,18], neurodegenerative diseases [19], neurological [20], neurodevelopmental [21], and psychiatric disorders [22]. 

These extracellular miRNAs can be categorized into two different types—either encapsulated in extracellular vesicles (EVs), including exosomes and microvesicles, or outside of EVs, which are usually complexed with proteins, such as lipoproteins and ribonucleoproteins [23]. Some EVs are revealed to be involved in cell-cell communication regulating physiological processes including immune response and neuronal activities [24,25]. Moreover, in several pathological conditions, EVs have been found to carry pathogenic proteins such as α-synuclein, β-amyloid, and prion proteins that can contribute to the pathogenesis and intercellular spread of diseases [26]. Therefore, EVs in body fluids are suggested to reflect status of their parental cells and are an effective source of biomarkers for liquid biopsy [27]. Indeed, previous studies have shown that EV-incorporated miRNA showed different profiles from outside of EVs [23,28,29]. In addition, some miRNAs in EVs showed a more consistent diagnostic value than whole plasma or urine for prostate cancer and preterm labor or type 1 diabetes [23,28,29]. Thus, isolating EVs from whole fluids and investigating the composition of miRNAs in EVs may provide different disease-associated signatures from the whole fluid or EV-depleted fraction. 

There have been studies on the involvement of miRNAs in PTSD using both animal models and patient samples. For example, in a rat learned helplessness stress PTSD model, miRNAs involved in fear response gene regulation showed similar changes in serum and amygdala [30]. Using a social defeat mouse PTSD model, we previously proposed miR-29 as one of the key regulators involved in PTSD associated heart pathologies by modulating extracellular matrix remodeling genes [31]. In combat veterans with PTSD, dysregulated miRNAs in peripheral blood mononuclear cells (PBMCs) were identified and they were associated with immune functions [32]. Another study using whole blood samples from military combat veterans with PTSD observed changes in miRNAs that regulate neuronal activities [33]. A whole blood miRNA study from the Grady trauma project showed two miRNAs, miR-212-3p and miR-3130-5p, implicated in neurological disorders were decreased in PTSD patients [34]. However, PTSD associated changes in extracellular miRNAs incorporated in EV or outside of EV in blood plasma have not been studied. 

In the present study, we hypothesized that in addition to whole plasma, characterizing miRNAs in EV and EV-depleted (EVD) plasma separately would lead to the identification of more informative PTSD-associated cell-free miRNAs. We profiled miRNAs in whole plasma, EVs and EVD plasma, and showed the distribution of miRNAs are different between EV and EVD plasma. We also identified PTSD-affected concentration changes in some miRNAs. We further validated the concentration changes of two miRNAs from EV (miR-203a-3p) and EVD plasma (miR-339-5p) in an independent cohort. Functional enrichment analyses of miRNA target genes suggested these two miRNAs are involved in signaling pathways and comorbid conditions (e.g., dopaminergic and serotonergic signaling, immune response, and cardiovascular and metabolic diseases) associated with PTSD.

## 2. Materials and Methods

### 2.1. Study Subjects

The study subjects consisted of 22 male PTSD patients and 22 age-, BMI-, and ethnicity-matched controls. This cohort is a subset of the male veterans from Operation Enduring Freedom (OEF) and Operation Iraqi Freedom (OIF) recruited by the New York University Langone Medical Center (NYULMC)/NYU School of Medicine (NYUSM) and the James J. Peters Veterans Affairs Medical Center (JJPVAMC)/Icahn School of Medicine at Mount Sinai (ISMMS) as described in Hammamieh et al. [35]. The detailed recruitment and assessment procedures were described in some previous studies [36,37]. In brief, subjects were interviewed by a clinical psychologist using the Clinician Administered PTSD Scale (CAPS) for Diagnostic and Statistical Manual of Mental Disorders 4th Edition (DSM-IV). PTSD diagnosis was based on CAPS criteria for DSM-IV with the additional provision that cases were included if they met criteria for current warzone related PTSD with current CAPS total scores greater than or equal to 40. Controls were negative for lifetime warzone and non-warzone PTSD and had current CAPS scores less than or equal to 20. None of the subjects met DSM-IV criteria for alcohol dependence within the past 8 months, drug abuse or dependence within the past year, or any psychiatric disorder with psychotic features, bipolar disorder, or obsessive-compulsive disorder.

### 2.2. EV Isolation and RNA Extraction from Plasma

Blood was drawn from each participating veteran in the morning after a night of fasting. Whole blood was collected into 10 mL vacutainer ethylenediaminetetraacetic acid (EDTA) tubes (Becton Dickinson, Franklin Lakes, NJ, USA). Plasma samples were prepared after removing blood cells by centrifugation for 10 min at 1100× *g*, aliquoted, and stored at −80 until subsequent use. To remove cell debris and platelets, plasma was centrifuged for 10 min at 10,000× *g*. EVs in plasma samples were isolated using size-exclusion chromatography as previously described [28]. In brief, 100 μL of plasma was added to the PBS equilibrated column (iZON science, Cambridge, MA, USA) and the sample was fractionated in 500 μL increments with 15 mL of PBS. Fractions 7–9 were combined as the EV fraction, and fractions 12–32 were combined as the depleted fraction. Both the EV and EV-depleted fractions were concentrated using an Amicon Ultra-4 10 K centrifugal filter (EMD Millipore, Billerica, MA, USA). Total RNA, including miRNA, was isolated from all three fractions (whole plasma, EV and EV-depleted) using a modified Qiagen miRNeasy Micro procedure (Qiagen, Germantown, MD, USA). The RNA quality and the concentration was assessed using an RNA Pico assay on an Agilent Bioanalyzer (Santa Clara, CA, USA).

### 2.3. Sequencing Library Construction

Small RNA sequencing libraries were constructed using a modified Illumina small RNA sequencing library construction method [38]. Briefly, T4 RNA ligase enzymes were used to attach Illumina compatible adapter sequences to the 3’ and 5’ ends of the RNA. These adapters each contain four degenerate bases at the RNA-adapter ligation site. The adapters were purchased from Integrated DNA Technologies (Coralville, IA, USA). Following adapter ligation, the libraries were incubated with a single-stranded binding protein (Promega, Madison, WI), 5’-deadenylase (New England Biolabs, Ipswich, MA, USA), and RecJf exonuclease (NEB) to reduce adapter-dimer formation. Then, cDNA was synthesized using SuperScript III reverse transcriptase (Invitrogen, Waltham, MA, USA) and the small RNA library was amplified by PCR (polymerase chain reaction) with a high-fidelity DNA polymerase (New England Biolabs, Ipswich, MA, USA), an Illumina universal primer, and selected indexed-primers. PCR products were cleaned with Ampure XP beads (Beckman Coulter, Indianapolis, IN, USA) and the library was size-selected using a PippinHT instrument (Sage Science, Beverly, MA, USA). The target range was set at 134–162 nt to recover inserts 20–22 nt in length. 

### 2.4. Library Sequencing

Small RNA libraries were quantified by quantitative realtime polymerase chain reaction (qRT-PCR) using the NEBNext Library Quant Kit for Illumina (NEB). An equimolar amount of each library was pooled and the final pooled concentration was determined by Qubit (Thermo Fisher Scientific, Waltham, MA, USA). Libraries were sequenced for 83 cycles on an Illumina NextSeq500 instrument (San Diego, CA, USA). BCL files were generated and then de-multiplexed to FASTQ files through Basespace (Illumina, San Diego, CA, USA) allowing zero mismatch for barcode sequence.

### 2.5. Small RNA Sequencing Data Analysis

All the sequencing data were processed and analyzed by sRNAnalyzer—a small RNA sequencing data analysis pipeline with default settings [39]. In brief, adapter sequences were trimmed with cutadapt [40]. Low-quality sequences were removed with Prinseq [41], and identical reads after trimming were collapsed with fastx_collapser. The preprocessed reads were mapped to various human reference databases including miRBase 21 [42] for microRNA, piRBase 1.0 [43] for piwiRNA, snoRNABase version 3 [44] for snoRNA, LNCipedia [45] for lncRNA, RepBase 19.10 [46] for repetitive sequences, RefSeq/NCBI for transcriptome sequences, Ensembl/EBI [47] for noncoding RNA (ncRNA), and finally human genome GRCh38. The miRDeep2 program was applied to identify novel miRNAs with default parameters [48]. Low abundant miRNAs with fewer than five mapped reads in less than 50% of samples were removed prior to normalization. The read counts were normalized by the TMM method [49]. Then, the abundance changes of miRNAs were identified using edgeR [50]. The miRNAs affected by PTSD were selected as *p* < 0.05 and fold-changes >1.5. 

### 2.6. qRT-PCR

Some of the miRNA concentration differences determined by NGS were also assessed by using the TaqMan Advanced miRNA Assay kit. In brief, 2 uL of isolated RNA from individual samples was reverse transcribed using the TaqMan advanced assay kit (Thermo Fisher, Waltham, MA, USA). Quantitative Polymerase Chain Reaction (qPCR) assay was performed on the BioRad CFX96 Touch thermocycler. For PCR assays, AmpliTaq Fast DNA Polymerase was activated at 95 °C for 20 s followed by 40 cycles of 2-step amplification at 95 °C for 3 s and 60 °C for 30 s. Data was acquired following the 30 s anneal/extension step at 60 °C. The reference miRNAs were selected from the sequencing data. First, miRNAs with fold-change <1.1, *p*-value > 0.05, and coefficient of variation (CV) < 0.05 in both PTSD- and PTSD+ samples were selected in whole plasma, EV, and EV-depleted plasma fractions. The miRNAs that met the criteria were then sorted by abundances. Then 4 miRNAs, hsa-miR-21-5p, hsa-miR-484-5p, hsa-miR-423-5p, and hsa-miR-140-3p, were selected and used to normalize all sample types. The normalization reference was determined as the geometric mean of the 4 miRNAs. Welch’s T-test was applied on the normalized qPCR data.

### 2.7. Functional Association of miRNA Target Genes

The target genes of the affected miRNAs were retrieved from miRTarBase, an experimentally validated miRNA-target interactions database [51]. To identify the biological processes of the miRNA target genes, we performed functional enrichment analyses of gene ontology biological processes (GOBPs) and Kyoto encyclopedia of genes and genomes (KEGG) pathways using the DAVID (database for annotation visualization and integrated discovery) [52]. The terms with *p*-value < 0.1 (default cutoff) and ≥3 target genes were selected as significantly enriched GOBPs or pathways by the target genes. To evaluate significance of the selected GOBPs and KEGG pathways, all miRNA-target gene interactions were retrieved from miRTarBase database and fold enrichment scores (FES) were calculated. The FES measures how strongly a miRNA is associated with a specific pathway. The FES were calculated as the ratios between (a/b) and (c/d) where “a” is the number of mRNA targets by a specific miRNA and involved in a specific pathway, “b” is the number of all mRNAs that can be targeted by the specific miRNA and involve in any biological process, “c” is the number of all genes involved in a specific pathway, and “d” is the total number of genes involved in biological processes or pathways included in the DAVID database. 

### 2.8. Ethics

This study was approved by the Institutional Review Boards of the New York University Langone Medical Center (New York, NY, USA), the Icahn School of Medicine at Mt Sinai (New York, NY, USA) and the James J Peters Veterans Administration Medical Center (Bronx, NY, USA) and was conducted in accordance with the Declaration of Helsinki. All subjects gave written informed consent to participate in the study.

## 3. Results

### 3.1. Demographic and Clinical Characteristics of Study Subjects

The demographic and clinical characteristics of the study participants are summarized in Table 1. Ethnicity and education level were similar between patients and controls. As expected, the symptom scales, including the CAPS, Symptom Checklist-90 (SLC-90) depression subscale and the Beck Depression Inventory II in PTSD positive were higher than in PTSD negative.

### 3.2. The Distribution of Small RNA in Circulation 

**Figure 1:**
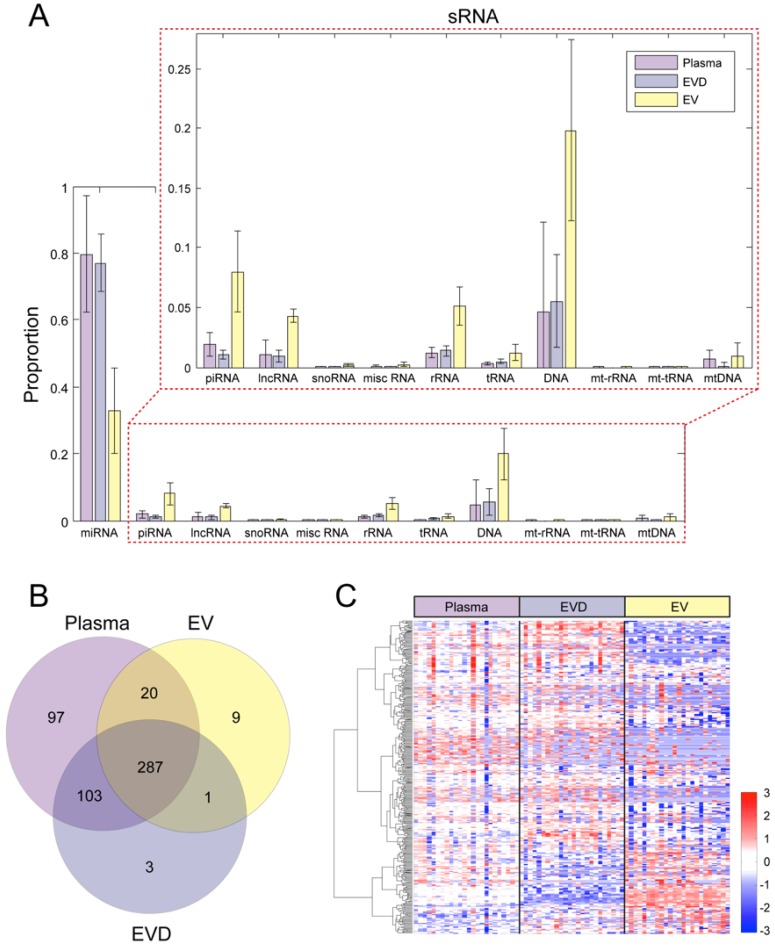
Different small RNA profiles in plasma, EV, and EVD samples. (**A**) The average small RNA compositions of the 24 subjects. (**B**) The number of the detected microRNAs (miRNAs) in each sample type. (**C**) The abundance profiles of the 287 miRNAs detected in all sample type.

Small RNA profiles were obtained from whole plasma, EV, and EVD plasma. As expected, miRNA accounted for the highest proportion of all mapped reads. MiRNAs occupied about 80% of the total mapped reads in whole plasma and EVD plasma samples, but only 33% in EV. In order to explain such difference in EV, the length distribution of the sequenced reads in whole plasma, EV and EVD plasma was examined. A much higher proportion of 22 nt reads that correspond to miRNA were observed in whole plasma and EVD plasma than in EV (Figure S1). The EV fraction had shorter (less than 20 nt) or longer (larger than 24 nt) reads than whole plasma and EVD plasma. This implies EVs may contain a different spectrum of RNAs than whole plasma or EVD plasma. Therefore, the abundance and distribution of different RNA types including miRNA, piwi-interacting RNA (piRNA), long non-coding RNA (lncRNA), small nucleolar RNA (snoRNA), miscellaneous RNA (misc RNA), ribosomal RNA (rRNA), and transfer RNA (tRNA) were compared in each sample type (Figure 1A). Compared to plasma and EVD, EV has higher proportion of other RNA types including piRNA, lncRNA, rRNA, and tRNA (*p*-value <1 × 10^−4^). As shown in Figure S2, the reads shorter or longer than 22 nt were mapped to other types of RNAs.

### 3.3. Circulating miRNAs Affected by PTSD 

On average, the number of observed miRNAs in whole plasma, EV, and EVD were 680, 453, and 551, respectively. A total of 2,751 miRNAs were detected in at least one sample used in this study. In order to identify reliably detected miRNAs, we selected miRNAs with ≥5 mapped reads in more than 50% of samples. Based on the aforementioned threshold, we obtained 507, 317, and 394 miRNAs in whole plasma, EV, and EVD plasma respectively (in total, 520 unique miRNAs were reliably detected in all sample types; Figure 1B). Most of the miRNAs that were detected in EV and EVD were also detected in whole plasma. Among them, 287 miRNAs were quantified in all three sample types (whole plasma, EV and EVD plasma). Most of the 287 common miRNAs had similar abundances in whole plasma and EVD plasma. However, EVs showed distinctive abundance profiles from plasma and EVD plasma samples (Figure 1C). Comparing EV and EVD samples, 89 miRNAs were enriched in the EV fractions while 78 miRNAs were underrepresented in EV. We searched for two known sequence motifs, GGAG or GGCU at 3’ end of miRNA that were reported for EV miRNA packaging [53]; however, these motifs were not observed in EV-enriched miRNAs. This suggests there might be other mechanisms involved in sorting miRNA into EVs. 

**Figure 2:**
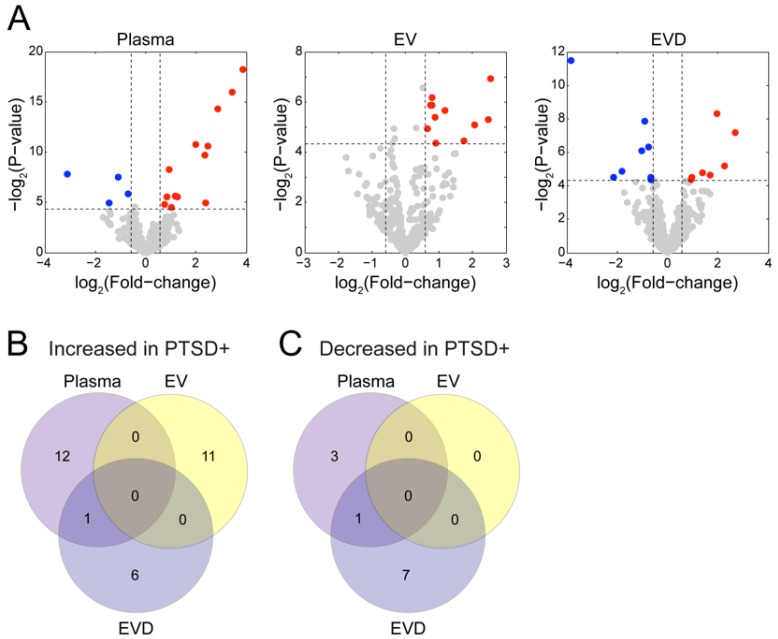
Differential abundances of the miRNAs between post-traumatic stress disorder- (PTSD-) and PTSD+ subjects in three fractions. (**A**) Red and blue dots indicate higher- and lower-abundance miRNAs in PTSD+ subjects. (**B**) The number of the increased miRNAs in PTSD+ subjects. (**C**) The number of the decreased miRNAs in PTSD+ subjects.

From the differential analysis, we identified 41 PTSD associated miRNA—17 (13 increased- and four decreased-concentrations) in whole plasma, 11 (all increased concentration in PTSD) in EV, and 15 (7 increased- and eight decreased-concentrations) in EVD plasma samples (Figure 2A). Each sample type showed distinctive miRNA concentration changes associated with PTSD. There were only two overlapping miRNAs affected by PTSD between whole plasma and EVD plasma samples, hsa-miR-7706-3p (increased concentration) and hsa-miR-143-3p (decreased concentration) in PTSD+ samples (Figure 2B,C). 

We further searched for novel putative miRNAs and found 46, 39 and 42 in whole plasma, EV and EVD plasma, respectively (Table S1). More than 60% of the identified novel miRNAs were detected in at least two sample types (Figure S3A). The numbers of novel miRNAs showing concentration changes in PTSD were small: one, five, and two in whole plasma, EV and EVD, respectively. No novel miRNA was affected by PTSD in all three different sample types (Figure S3B).

### 3.4. Other Types of Small RNAs Affected by PTSD 

We also analyzed the effect of PTSD on other types of small RNAs in circulation, specifically piRNA and snoRNA. The numbers of reliably detected piRNAs were much smaller than miRNAs; 61 in whole plasma, 58 in EV, and 48 in EVD plasma (Table S2). While 85% (41 out of 48) of the detected piRNAs in EVD were also identified in plasma, 17% (10 out of 58) of piRNAs were uniquely detected in EV (Figure S4A). We observed two, six, and one piRNAs that showed PTSD-associated concentration changes in whole plasma, EV, and EVD (Table S2). Interestingly, five piRNAs among the 10 uniquely detected in EV showed abundance changes in PTSD patients (Figure S4B). The numbers of reliably detected snoRNAs were even lower than piRNAs; 16 in whole plasma, two in EV, and seven in EVD (Table S3). All the detected snoRNAs in EV and EVD were also detected in whole plasma. There was no snoRNAs that showed concentration changes in PTSD patients (Figure S5).

### 3.5. Validation of the miRNA Associated with PTSD Using qRT-PCR

**Figure 3:**
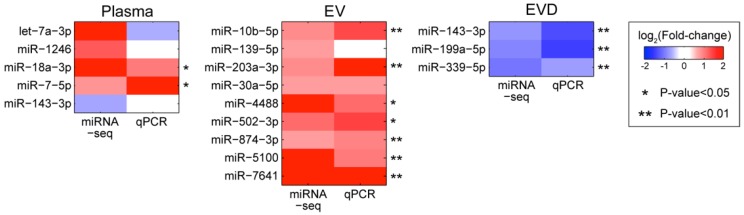
Consistent differential abundances of the miRNAs in qPCR and sequencing platforms in the discovery set. The color gradient indicates the fold changes between the PTSD- and PTSD+ subjects.

**Figure 4:**
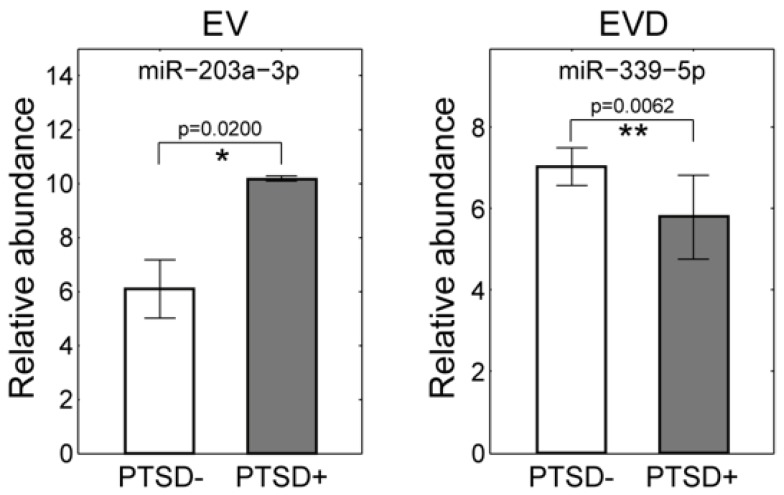
The confirmed differential abundances of miR-203a-3p and miR-339-5p in the independent validation set. The p-values were calculated by unadjusted T-test. * *p*-value < 0.05; ** *p*-value < 0.01.

We further tested the concentration changes of miRNA associated by PTSD using qRT-PCR. We selected 16 affected miRNAs which included four miRNAs in whole plasma, nine in EV, two in EVD, and one overlapping affected miRNA between whole plasma and EVD samples based on abundances, fold-changes, and availability of commercial assay. We first assessed the concentration changes of those 16 selected miRNAs with qRT-PCR using pooled samples that were used for the sequencing analysis (Figure 3). As shown in Figure 3, the qRT-PCR results showed a good agreement with the sequencing results, especially in EV and EVD fractions. Among the 16 tested miRNAs, 12 of them were confirmed with qRT-PCR including two from whole plasma (miR-18a-3p and miR-7-1-5p), seven from EV (miR-10b-5p, miR-203a-3p, miR-4488, miR-502-3p, miR-874-3p, miR-5100, miR-7641), and two from EVD plasma (miR-199a-5p and miR-339-5p) and one overlapped between whole plasma and EVD (miR-143-3p). In summary, most of the changes in the level of miRNAs in EV (seven out of nine) and EVD (three out of three) were validated by qRT-PCR. The 12 confirmed miRNAs were further tested in an independent cohort consisting of 10 PTSD negative (control) and 10 PTSD positive subjects. Two PTSD-associated miRNAs, miR-203a-3p (increased level in the EV) and miR-339-5p (decreased level in EVD plasma), were validated in the independent cohort (*p*-value < 0.05 and <0.01 from unadjusted T-test) (Figure 4).

### 3.6. Biological Processes May Be Affected by PTSD-Associated miRNA

**Figure 5:**
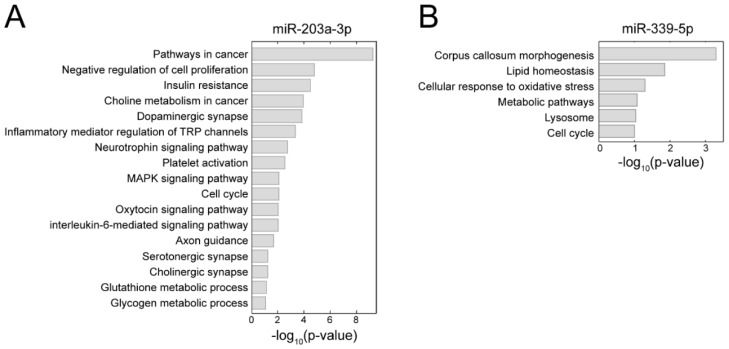
Functional association of miR-203a-3p and miR-339-5p. Gene ontology biological processes (GOBPs) and Kyoto encyclopedia of genes and genomes (KEGG) pathways of the target genes of (**A**) miR-203a-3p and (**B**) miR-339-5p.

In order to infer the biological functions of the two validated miRNAs, we first obtained their target genes from the miRTarBase, which provides experimentally validated miRNA-target interactions. Three hundred and eight genes were found as miR-203a-3p target genes. Those 308 genes were associated with synapse, various signaling pathways, metabolism, inflammation, cell cycle, and platelet activation (Figure 5A). MiR-339-5p had 235 target genes and they were associated with corpus callosum morphogenesis, lysosome, lipid homeostasis, metabolic process, oxidative stress, and cell cycle (Figure 5B). In order to evaluate significance of these GOBPs and pathways, we computed fold enrichment scores (FES) of all other miRNAs that have no evidence of being altered in PTSD for these GOBPs and pathways. Among the 2597 miRNAs that were included in the database and had no evidence of alteration between groups, less than 5% of them showed higher FES than the two validated miRNAs for most of the GOBPs and pathways (Table S4). Therefore, it is unlikely that these pathways were identified by chance.

## 4. Discussion

In this study, we characterized the circulating miRNA profiles in whole plasma, EV and EV-depleted plasma (EVD) fractions. To the best of our knowledge, this is the first comprehensive characterization of circulating miRNAs in plasma, EV and EVD plasma in participants with PTSD. Our sequencing results showed a distinct RNA spectrum in EV, a lower overall concentration of miRNA and higher percentages of other RNA types including piRNAs and snoRNAs in EV, compared to whole plasma and EVD. Of the 2751 miRNAs observed in at least one sample used in this study, 520 of them were reliably quantified. Among them, the concentrations of several miRNAs and piRNAs were affected by PTSD. We confirmed the concentration changes for most of the PTSD associated miRNAs, especially in EV and EVD, with qRT-PCR in the same discovery cohort. Two of the miRNAs, miR-203a-3p in EV and miR-339-5p in EVD were further validated in the independent cohort. Therefore, EV can provide unique pathophysiological information and may be a better source of biomarkers for PTSD compared to whole plasma.

The miRNA miR-203a-3p has been shown to be involved in synapse, central nervous system (CNS) development and function, and glioblastoma [54,55,56]. It is also known to regulate inflammatory mediator production, such as IL-6 and IL-8, and induces inflammatory response [57,58,59]. In line with this, our analysis revealed that miR-203a-3p regulates genes involved in the neurotransmitter system (e.g., cholinergic, dopaminergic and serotonergic systems), neural development (e.g., neurotrophin signaling pathway and axon guidance), and immune response (e.g., interleukin-6-mediated signaling pathway) which have been reported as being altered in PTSD [60,61,62,63]. In addition, miR-203a-3p affects various pathways involved in comorbid conditions of PTSD such as cancer (e.g., pathways in cancer), cardiovascular disease and cardiac infarction (e.g., platelet activation), diabetes and metabolic disease (e.g., insulin resistance) (Figure 5A) [64,65,66]. 

PTSD has been suggested as a risk factor for dementia [67] and miR-339-5p is known to be associated with neurodegenerative disorders. MiR-339-5p is also involved in brain inflammation [68,69,70]. It plays a role in corpus callosum morphogenesis where the area and volume are reduced in some PTSD patients [71,72,73]. MiR-339-5p is also associated with lysosome, lipid homeostasis and oxidative stress that have been shown to be altered in PTSD [69,74]. 

In addition to miRNAs, we analyzed other types of small RNAs, specifically piRNAs and snoRNAs. The number of detectable piRNAs and snoRNAs were much smaller than miRNAs. In addition, there were only a few piRNAs or snoRNAs that showed differential expression in PTSD. Our small RNA library preparation method selectively enriches fragment size for 20–22 nt which may contribute to the low detection rate of longer RNAs. Since the length of piRNAs (26–31 nt) and snoRNAs (60–300 nt) is longer than miRNA (21–24 nt) and is outside of our library size selection range, the profile of piRNAs and snoRNAs probably is not from intact forms and the reads may come from degraded forms. Other types of small RNAs including piRNAs and snoRNAs also have been observed in body fluids [75,76]. A few studies on the spectrum changes of piRNAs and snoRNAs have been reported in cancers, Alzheimer’s disease, and acute CNS injuries [77,78,79]; however, studies on circulating piRNAs and snoRNAs are limited [80,81]. Complete characterization of total RNA transcriptome may provide additional PTSD-associated signatures [82]. 

The molecular content, including miRNAs, in EVs can be transferred to and affect the phenotype of recipient cells. For example, it has been shown that intercellular transfer of miRNAs via EVs to neurons and glia affects the development, homeostasis, and pathological states of CNS [83]. Therefore, EVs may be involved in systemic pathophysiology [84]; however, to understand EV-mediated systematic signaling, it is critical to be able to analyze the molecular content, determine the origin of EV, and identify the targeted organs. Even though we may not be able to precisely determine the origin of EVs in circulation, there are examples that EV surface markers can be used to assess the origin of EVs [85]. Whole-body intravital imaging has been used to track the distribution of fluorescent dye labeled EVs in a mouse model [86]. Therefore, it is feasible to conduct translational studies to determine the target organ of EVs and assess the functionality of specific miRNAs encapsulated in EV in an animal model. Results from animal studies may provide more specific EV-mediated biological mechanisms on how the complex interactions between different organs can occur and affect each other in complex diseases like PTSD. In addition, being able to identify the origin of EVs might assist the identification of biomarkers that differentiate closely related disorders since they may involve different cell types. For example, previous research has shown that the release of EVs was dysregulated depending on pathologic conditions, e.g., increased EV release from neurons of the frontal cortex of Down syndrome patients and hippocampus of a mouse model subjected to traumatic brain injury [87,88]. Therefore, additional studies on stoichiometry of the rate of EV-release by various tissues into plasma might help to develop more specific biomarkers diagnosing diseases with overlapping symptoms. The isolation of EVs from body fluids requires additional time and resources; however, rapid advances in EV characterization techniques and isolation from body fluids will lead to fast, sensitive, and cost-effective detection of EV-associated biomarkers in the near future [89,90,91].

There are limitations in our study. Due to the small sample size, our results should be considered as an exploratory study and the results should be validated in a large independent cohort. Additionally, our study included only male veterans, the differential abundances of the two miRNAs should also be investigated in female cohorts. Establishing a PTSD biobank by recruiting participants from diverse populations in collaboration with multiple institutions would be a useful resource to validate the miRNAs identified as existing biobanks for other psychiatric disorders [92]. Because we studied veterans with chronic PTSD in a cross sectional design we do not know if the changes in miRNAs pre-existed the development of PTSD, serving as risk factors, or represent illness markers. Measuring abundances of the two miRNAs over the course of the development of PTSD in a longitudinal study might help to confirm if they are risk factors or markers for PTSD. Additionally, studying relationships between abundances of the two miRNAs in the course of treatment may be useful to evaluate its application as markers for therapeutic efficacy. Lastly, as recent studies demonstrated, other types of measurements such as serum proteins and heart rate dynamics have diagnostic power and integrating different information can increase classification accuracy [93,94]. Therefore, circulating miRNAs reported in this study may gain additional diagnostic accuracy when combined with other measurement results.

## 5. Conclusions

This study presents the results from a comprehensive analysis of miRNA profile changes in whole plasma, EV and EV-depleted plasma. The results suggest that separating EVs from whole plasma provides additional altered molecular profiles in PTSD. Our comprehensive analyses and validation experiments identified two PTSD-associated miRNAs from EV and EV-depleted plasma fractions. Further study with independent populations will be required to evaluate the clinical use of these miRNAs in PTSD.

## Figures and Tables

**Table 1:** Demographic and clinical characteristics.

Cohort	Discovery Set				Validation Set			
PTSD Status	PTSD-		PTSD+		PTSD-		PTSD+	
	Mean	SD	Mean	SD	Mean	SD	Mean	SD
Age (years)	34.08	10.03	30.50	3.55	31.3	6.15	31.1	2.85
BMI (kg/m^2^)	26.13	2.45	27.13	3.24	26.7	3.66	28.03	4.40
Ethnicity N (%) of Non-Hispanic White	4 (33.3)		6 (50.0)		4 (40.0)		5 (50.0)	
Education	3.58	1.16	3.25	0.87	4.00	0.67	3.60	1.07
CAPS Total Score life time	0.42	1.44	81.17	12.26	4.6	5.87	91.2	14.54
SCL90 Depression	0.27	0.67	1.82	0.62	0.16	0.22	2.11	0.95
Beck Depression Inventory II	3.25	6.37	26.17	8.02	1.33	2.00	27	9.13

## Supplementary Materials

The following are available online at https://www.mdpi.com/2077-0383/8/7/963/s1, Figure S1: The read length distribution in each sample type, Figure S2: The distribution of mapped read length, Figure S3: The number of the detected novel miRNAs in each sample type, Figure S4: The number of the detected piRNAs in each sample type, Figure S5: The number of the detected snoRNAs in each sample type, Table S1: The list of detected novel miRNAs, Table S2: The list of detected piRNAs, Table S3: The list of detected snoRNAs, Table S4: The list of GOBPs and KEGG pathways of the target genes of miR-203a-3p and miR-339-5p.
