# Supplementary material for: Distinct Profiles of Cell-Free MicroRNAs in Plasma of Veterans with Post-Traumatic Stress Disorder
Source: J Clin Med. 2019 Jul 3;8(7):963. doi: 10.3390/jcm8070963 (PMC6678393; doi:10.3390/jcm8070963)

List of supplementary material

Table S1. The list of detected novel miRNAs. The novel miRNAs that had significantly higher or lower abundance in PTSD+ patients were marked with + and -, respectively.

Table S2. The list of detected piRNAs. The piRNAs that had significantly higher or lower abundance in PTSD+ patients were marked with + and -, respectively.

Table S3. The list of detected snoRNAs.

Table S4. The list of GOBPs and KEGG pathways of the target genes of miR-203a-3p and miR-339-5p. Count indicates the number of genes of the involved in the pathway targeted by the miRNA. FES indicates Fold Enrichment Score. % FES in the last column indicates the percentage of miRNAs not altered in PTSD that showed higher FES than miR-203a-3p or miR-339-5p.

Figure S1. The read length distribution in each sample type.

Figure S2. The distribution of mapped read length in A. plasma, B. EV, C. EVD.

Figure S3. The number of the detected novel miRNAs in each sample type.

Figure S4. The number of the detected piRNAs in each sample type.

Figure S5. The number of the detected snoRNAs in each sample type.

Table S1. The list of detected novel miRNAs. The novel miRNAs that had significantly higher or lower abundance in PTSD+ patients were marked with + and -, respectively.

No.	Plasma		EV		EVD	
1	novel:chr6_12380-5p	+	novel:chr1_658-5p	+	novel:chr12_3019-3p	+
2	novel:chr2_7249-3p		novel:chr6_12347-3p	+	novel:chr2_7720-5p	+
3	novel:chr6_12457-3p		novel:chr8_14037-3p	+	novel:chr2_7249-3p	
4	novel:chr10_1426-5p		novel:chr15_4656-5p	-	novel:chr7_12896-5p	
5	novel:chr10_1605-3p		novel:chr8_13987-3p	-	novel:chr1_586-5p	
6	novel:chr11_1960-5p		novel:chr10_1605-3p		novel:chr10_1605-3p	
7	novel:chr11_2062-5p		novel:chr14_3931-3p		novel:chr11_2062-5p	
8	novel:chr11_2086-5p		novel:chr2_7607-3p		novel:chr11_2086-5p	
9	novel:chr11_2101-3p		novel:chr4_10699-3p		novel:chr12_2936-5p	
10	novel:chr11_2427-3p		novel:chr6_12797		novel:chr12_3042-5p	
11	novel:chr12_2936-5p		novel:chr11_2086-5p		novel:chr13_3366-5p	
12	novel:chr12_3019-3p		novel:chr1_586-5p		novel:chr15_4574-3p	
13	novel:chr15_4656-5p		novel:chr10_1426-5p		novel:chr15_4656-5p	
14	novel:chr15_4697-3p		novel:chr11_2062-5p		novel:chr15_4697-3p	
15	novel:chr17_5687-3p		novel:chr12_3019-3p		novel:chr17_5687-3p	
16	novel:chr17_5852-5p		novel:chr12_3042-5p		novel:chr17_5852-5p	
17	novel:chr17_5857-3p		novel:chr13_3366-5p		novel:chr17_5857-3p	
18	novel:chr19_6455-3p		novel:chr14_3909-3p		novel:chr2_7004-3p	
19	novel:chr19_6786-5p		novel:chr15_4574-3p		novel:chr2_7826-5p	
20	novel:chr2_7004-3p		novel:chr15_4697-3p		novel:chr20_8297-3p	
21	novel:chr2_7607-3p		novel:chr16_4924-5p		novel:chr20_8485-3p	
22	novel:chr2_7720-5p		novel:chr2_7249-3p		novel:chr21_8737-5p	
23	novel:chr20_8297-3p		novel:chr2_7720-5p		novel:chr21_8881-3p	
24	novel:chr20_8485-3p		novel:chr4_10472-5p		novel:chr22_8993-3p	
25	novel:chr21_8737-5p		novel:chr5_11017-3p		novel:chr22_9166-3p	
26	novel:chr21_8816-5p		novel:chr5_11144-5p		novel:chr22_9170-3p	
27	novel:chr22_8993-3p		novel:chr5_11212-5p		novel:chr3_9461-3p	
28	novel:chr22_9166-3p		novel:chr5_11251-3p		novel:chr4_10472-5p	
29	novel:chr3_9461-3p		novel:chr5_11540-5p		novel:chr4_10699-3p	
30	novel:chr3_9508-5p		novel:chr6_12250-5p		novel:chr5_11017-3p	
31	novel:chr4_10472-5p		novel:chr6_12380-5p		novel:chr5_11264-5p	
32	novel:chr4_10699-3p		novel:chr6_12451-3p		novel:chr5_11628-5p	
33	novel:chr4_10797-3p		novel:chr7_12896-5p		novel:chr6_12250-5p	
34	novel:chr5_11017-3p		novel:chr8_13981-3p		novel:chr6_12347-3p	
35	novel:chr5_11264-5p		novel:chr9_14507-3p		novel:chr6_12380-5p	
36	novel:chr5_11628-5p		novel:chr9_14526-5p		novel:chr6_12451-3p	
37	novel:chr6_12250-5p		novel:chrX_15877-3p		novel:chr8_13987-3p	
38	novel:chr6_12347-3p		novel:chr1_658-5p		novel:chr8_14037-3p	
39	novel:chr6_12451-3p		novel:chr6_12347-3p		novel:chr9_14480-5p	
40	novel:chr7_12820-5p				novel:chr9_14507-3p	
41	novel:chr7_12877-3p				novel:chr12_3019-3p	
42	novel:chr7_12896-5p				novel:chr2_7720-5p	
43	novel:chr8_13981-3p					
44	novel:chr8_13987-3p					
45	novel:chr9_14480-5p					
46	novel:chr9_14507-3p					

Table S2. The list of detected piRNAs. The piRNAs that had significantly higher or lower abundance in PTSD+ patients were marked with + and -, respectively.

No.	Plasma		EV		EVD	
1	piR-hsa-5067	+	piR-hsa-14870	+	piR-hsa-29939	+
2	piR-hsa-793	+	piR-hsa-23533	+	piR-hsa-1043	
3	piR-hsa-1043		piR-hsa-23566	+	piR-hsa-1100	
4	piR-hsa-1100		piR-hsa-23672	+	piR-hsa-11256	
5	piR-hsa-1177		piR-hsa-29939	+	piR-hsa-1177	
6	piR-hsa-1207		piR-hsa-26925	-	piR-hsa-1207	
7	piR-hsa-12454		piR-hsa-1043		piR-hsa-1254	
8	piR-hsa-12487		piR-hsa-1079		piR-hsa-1282	
9	piR-hsa-1254		piR-hsa-1100		piR-hsa-1359	
10	piR-hsa-1282		piR-hsa-11256		piR-hsa-20266	
11	piR-hsa-1359		piR-hsa-1177		piR-hsa-20613	
12	piR-hsa-14870		piR-hsa-1207		piR-hsa-2107	
13	piR-hsa-1849		piR-hsa-1219		piR-hsa-21126	
14	piR-hsa-18905		piR-hsa-12454		piR-hsa-22381	
15	piR-hsa-20266		piR-hsa-12487		piR-hsa-23209	
16	piR-hsa-20613		piR-hsa-1254		piR-hsa-23210	
17	piR-hsa-21126		piR-hsa-12789		piR-hsa-23248	
18	piR-hsa-2117		piR-hsa-1282		piR-hsa-23317	
19	piR-hsa-2154		piR-hsa-1359		piR-hsa-23533	
20	piR-hsa-22381		piR-hsa-19620		piR-hsa-23638	
21	piR-hsa-23209		piR-hsa-20266		piR-hsa-23672	
22	piR-hsa-23210		piR-hsa-20613		piR-hsa-23919	
23	piR-hsa-23248		piR-hsa-2107		piR-hsa-25783	
24	piR-hsa-23317		piR-hsa-21126		piR-hsa-26589	
25	piR-hsa-23533		piR-hsa-23209		piR-hsa-26681	
26	piR-hsa-23555		piR-hsa-23210		piR-hsa-26747	
27	piR-hsa-23566		piR-hsa-23248		piR-hsa-26872	
28	piR-hsa-23617		piR-hsa-23317		piR-hsa-26925	
29	piR-hsa-23672		piR-hsa-23619		piR-hsa-27140	
30	piR-hsa-23919		piR-hsa-23638		piR-hsa-27282	
31	piR-hsa-24672		piR-hsa-23919		piR-hsa-27400	
32	piR-hsa-25783		piR-hsa-24684		piR-hsa-27616	
33	piR-hsa-26589		piR-hsa-25783		piR-hsa-27622	
34	piR-hsa-26681		piR-hsa-26508		piR-hsa-27728	
35	piR-hsa-26747		piR-hsa-26589		piR-hsa-28190	
36	piR-hsa-26872		piR-hsa-26747		piR-hsa-28212	
37	piR-hsa-26925		piR-hsa-27282		piR-hsa-28223	
38	piR-hsa-27007		piR-hsa-27400		piR-hsa-28374	
39	piR-hsa-27282		piR-hsa-27616		piR-hsa-28391	
40	piR-hsa-27400		piR-hsa-27622		piR-hsa-28467	
41	piR-hsa-27616		piR-hsa-27728		piR-hsa-28478	
42	piR-hsa-27622		piR-hsa-27731		piR-hsa-28489	
43	piR-hsa-27728		piR-hsa-28131		piR-hsa-28876	
44	piR-hsa-27731		piR-hsa-28190		piR-hsa-29114	
45	piR-hsa-28205		piR-hsa-28212		piR-hsa-30715	
46	piR-hsa-28212		piR-hsa-28223		piR-hsa-5067	
47	piR-hsa-28223		piR-hsa-28231		piR-hsa-5939	
48	piR-hsa-28374		piR-hsa-28374		piR-hsa-619	
49	piR-hsa-28391		piR-hsa-28391			
50	piR-hsa-28467		piR-hsa-28467			
51	piR-hsa-28478		piR-hsa-28478			
52	piR-hsa-28488		piR-hsa-28489			
53	piR-hsa-28634		piR-hsa-28841			
54	piR-hsa-28841		piR-hsa-28876			
55	piR-hsa-29114		piR-hsa-426			
56	piR-hsa-29939		piR-hsa-5939			
57	piR-hsa-30715		piR-hsa-619			

58	piR-hsa-3405		piR-hsa-793			
59	piR-hsa-3645					
60	piR-hsa-5939					
61	piR-hsa-619					

Table S3. The list of detected snoRNAs.

No.	Plasma	EV	EVD
1	ACA3-2	ACA45	ACA45
2	ACA33	U3-2B	HBI-100
3	ACA45		U26
4	E3		U3-2B
5	HBI-100		U33
6	U19		U65
7	U22		U92
8	U26		
9	U29		
10	U3-2B		
11	U31		
12	U33		
13	U65		
14	U74		
15	U92		
16	U95		

Table S4. The list of GOBPs and KEGG pathways of the target genes of miR-203a-3p and miR-339-5p. Count indicates the number of genes of the involved in the pathway targeted by the miRNA. FES indicates Fold Enrichment Score. %FES in the last column indicates the percentage of miRNAs not altered in PTSD that showed higher FES than miR-203a-3p or miR-339-5p.

ID	Term	Count	FES by **miR-203a-3p**	PValue	%FES
hsa05200	Pathways in cancer	29	3.89	0.0000	0.9241
GO:0008285	negative regulation of cell proliferation	21	3.09	0.0000	0.3851
hsa04931	Insulin resistance	11	5.37	0.0000	2.2333
hsa05231	Choline metabolism in cancer	10	5.22	0.0001	1.8868
hsa04728	Dopaminergic synapse	11	4.53	0.0001	1.4247
hsa04750	Inflammatory mediator regulation of TRP channels	9	4.84	0.0005	1.1937
hsa04722	Neurotrophin signaling pathway	9	3.96	0.0018	5.5834
hsa04611	Platelet activation	9	3.65	0.0030	4.5822
hsa04010	MAPK signaling pathway	12	2.48	0.0084	5.9684
hsa04110	Cell cycle	8	3.40	0.0086	10.3581
hsa04921	Oxytocin signaling pathway	9	3.00	0.0096	7.8552
GO:0070102	interleukin-6-mediated signaling pathway	3	19.44	0.0097	0.6161
GO:0007411	axon guidance	8	2.93	0.0196	1.3477
hsa04725	Cholinergic synapse	6	2.85	0.0577	5.6989
hsa04726	Serotonergic synapse	6	2.85	0.0577	5.0828
GO:0006749	glutathione metabolic process	4	4.16	0.0707	2.9650
GO:0005977	glycogen metabolic process	3	6.03	0.0874	3.8891

ID	Term	Count	FES by **miR-339-5p**	PValue	%FES
GO:0021540	corpus callosum morphogenesis	3	77.38	0.0005	0.2695
GO:0055088	lipid homeostasis	4	7.94	0.0136	1.8098
GO:0034599	cellular response to oxidative stress	4	4.84	0.0494	3.4655
hsa01100	Metabolic pathways	24	1.38	0.0830	2.6569
hsa04142	Lysosome	5	2.91	0.0899	1.8868
hsa04110	Cell cycle	5	2.84	0.0962	15.8260

Figure S1. The read length distribution in each sample type.

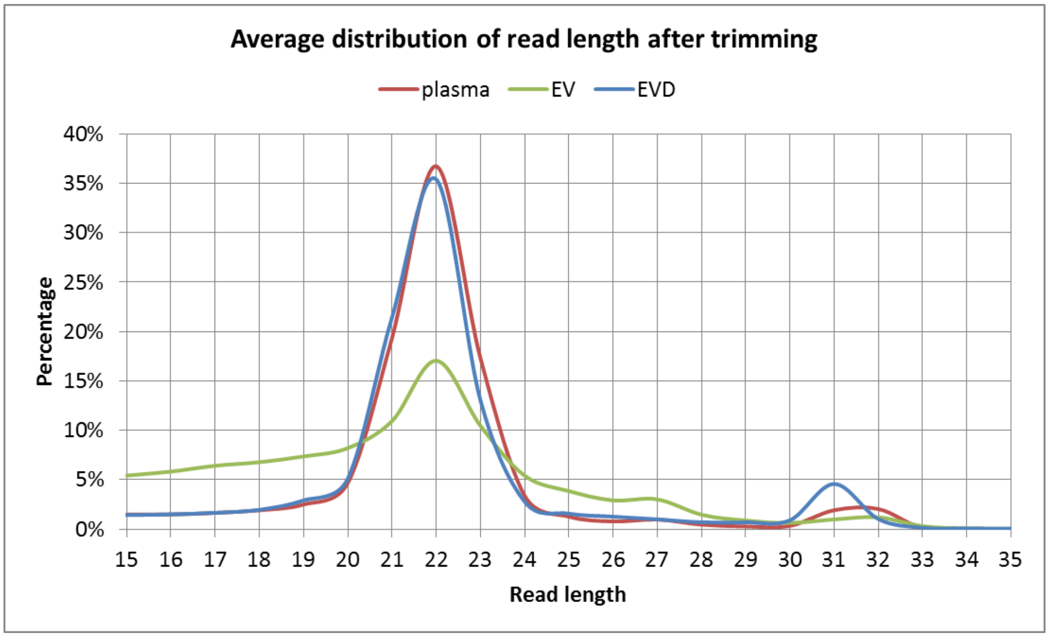

Figure S2. The distribution of mapped read length in A. plasma, B. EV, C. EVD.

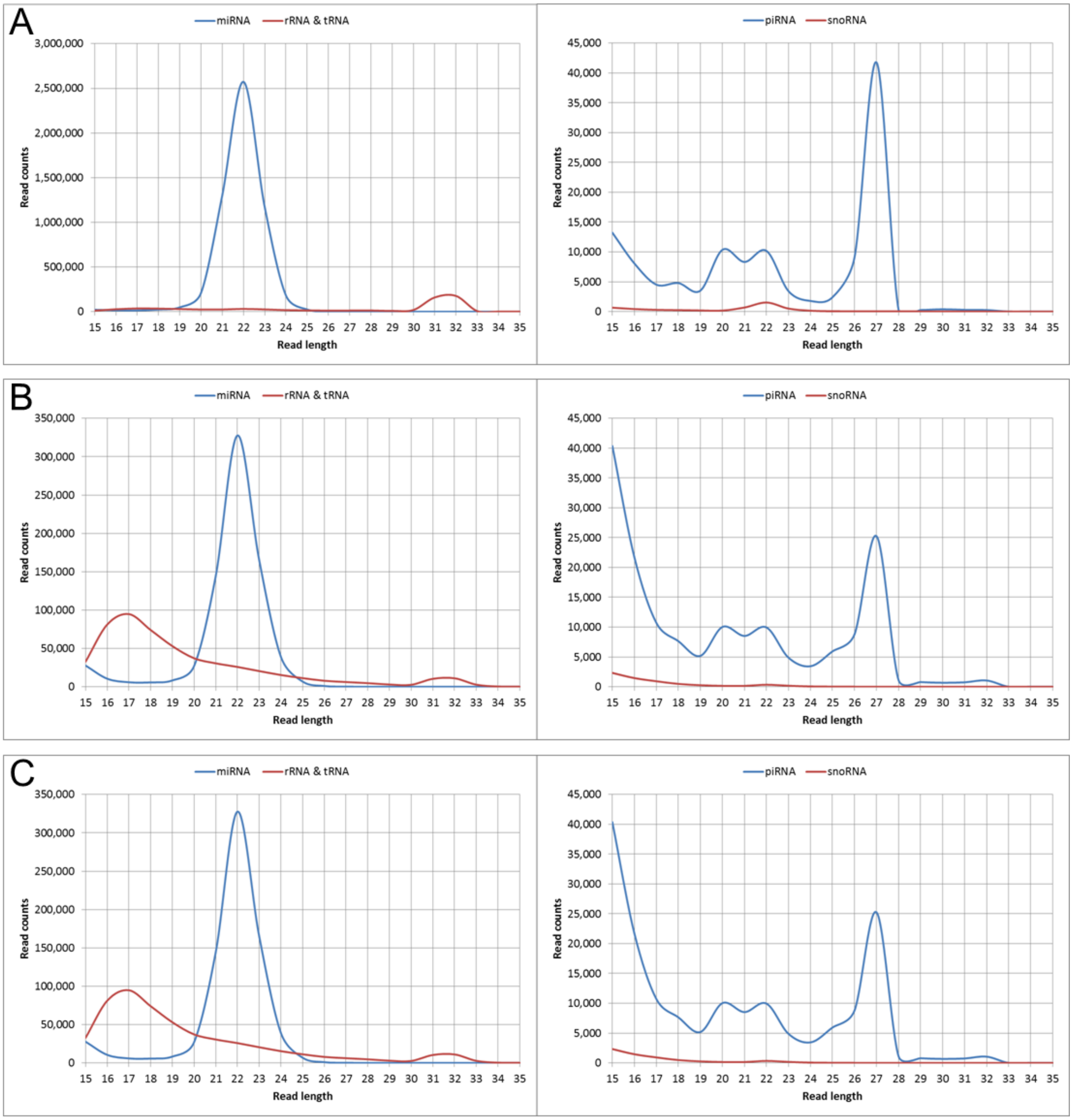

Figure S3. The number of the detected novel miRNAs in each sample type.

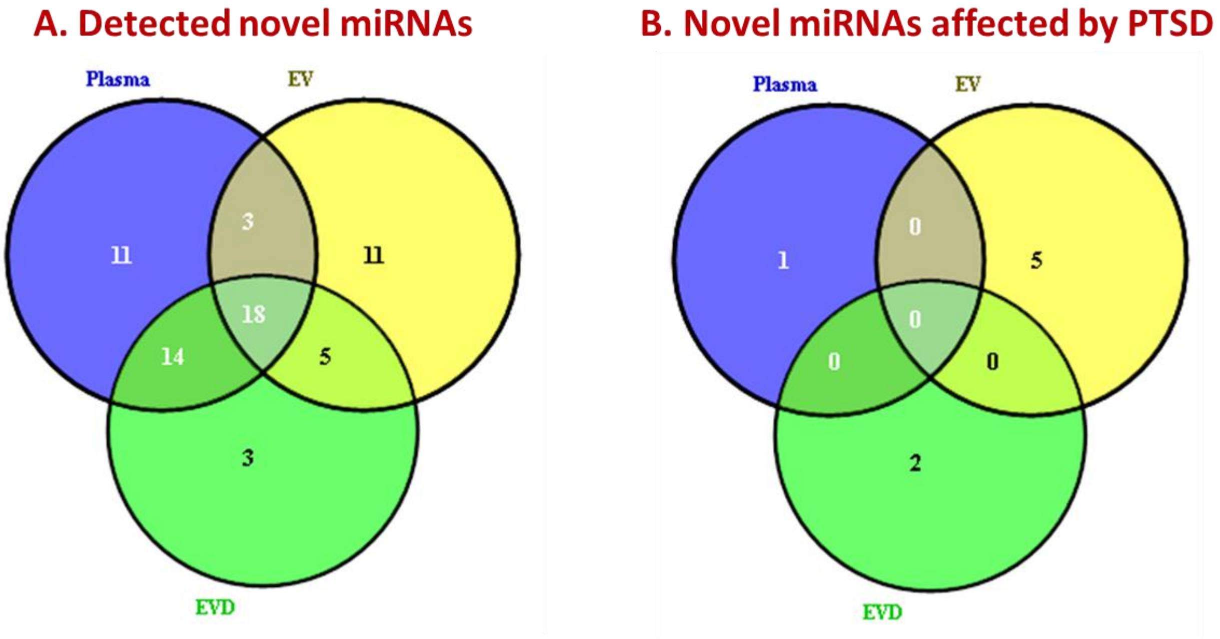

Figure S4. The number of the detected piRNAs in each sample type.

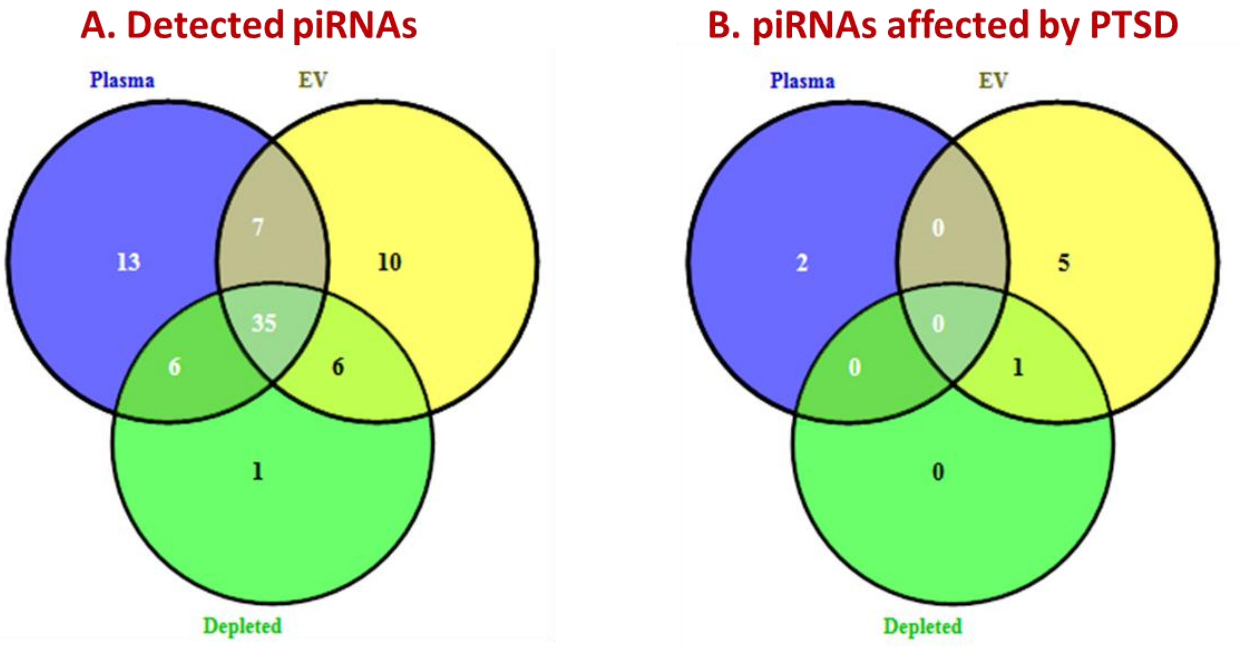

Figure S5. The number of the detected snoRNAs in each sample type.

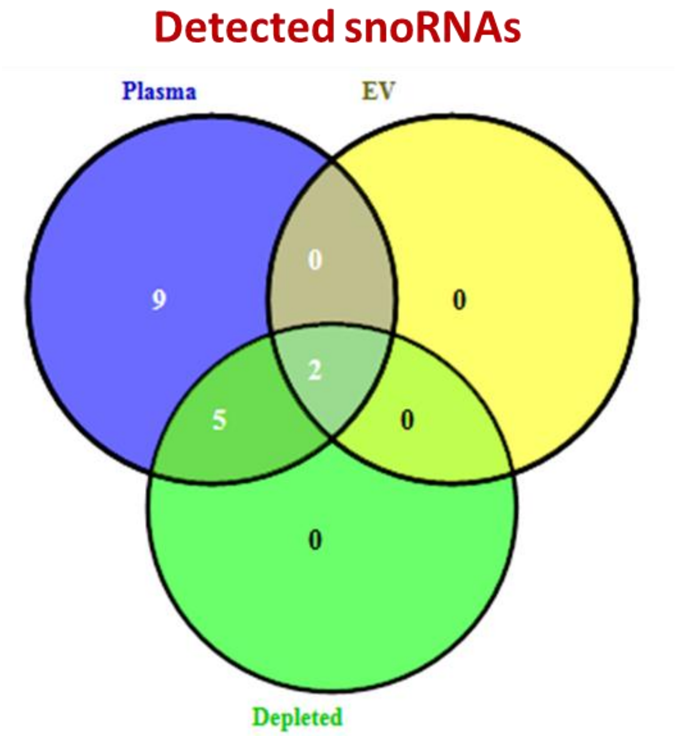

Supplement: Supplementary file 1 [file jcm-08-00963-s001.pdf]
